# Robot-assisted vs. sensory integration training in treating gait and balance dysfunctions in patients with multiple sclerosis: a randomized controlled trial

**DOI:** 10.3389/fnhum.2014.00318

**Published:** 2014-05-22

**Authors:** Marialuisa Gandolfi, Christian Geroin, Alessandro Picelli, Daniele Munari, Andreas Waldner, Stefano Tamburin, Fabio Marchioretto, Nicola Smania

**Affiliations:** ^1^Department of Neurological and Movement Sciences, Neuromotor and Cognitive Rehabilitation Research Center, University of VeronaVerona, Italy; ^2^Department of Neurological Rehabilitation, Private Hospital Villa MelittaBolzano, Italy; ^3^Neurology Section, Department of Neurological and Movement Sciences, University of VeronaVerona, Italy; ^4^Neurological Unit, Sacro Cuore–Don Calabria HospitalVerona, Italy; ^5^Neurological Rehabilitation Unit, Azienda Ospedaliera Universitaria IntegrataVerona, Italy

**Keywords:** sensory feedback, proprioception, postural balance, motor skills disorders, physiological adaptations

## Abstract

**Background:** Extensive research on both healthy subjects and patients with central nervous damage has elucidated a crucial role of postural adjustment reactions and central sensory integration processes in generating and “shaping” locomotor function, respectively. Whether robotic-assisted gait devices might improve these functions in Multiple sclerosis (MS) patients is not fully investigated in literature.

**Purpose:** The aim of this study was to compare the effectiveness of end-effector robot-assisted gait training (RAGT) and sensory integration balance training (SIBT) in improving walking and balance performance in patients with MS.

**Methods:** Twenty-two patients with MS (EDSS: 1.5–6.5) were randomly assigned to two groups. The RAGT group (*n* = 12) underwent end-effector system training. The SIBT group (*n* = 10) underwent specific balance exercises. Each patient received twelve 50-min treatment sessions (2 days/week). A blinded rater evaluated patients before and after treatment as well as 1 month post treatment. Primary outcomes were walking speed and Berg Balance Scale. Secondary outcomes were the Activities-specific Balance Confidence Scale, Sensory Organization Balance Test, Stabilometric Assessment, Fatigue Severity Scale, cadence, step length, single and double support time, Multiple Sclerosis Quality of Life-54.

**Results:** Between groups comparisons showed no significant differences on primary and secondary outcome measures over time. Within group comparisons showed significant improvements in both groups on the Berg Balance Scale (*P* = 0.001). Changes approaching significance were found on gait speed (*P* = 0.07) only in the RAGT group. Significant changes in balance task-related domains during standing and walking conditions were found in the SIBT group.

**Conclusion:** Balance disorders in patients with MS may be ameliorated by RAGT and by SIBT.

## Introduction

Multiple Sclerosis (MS) is a chronic disease of the central nervous system characterized by a progressive decline in various neurologic functions such as vision, sensation, coordination and balance, muscle strength and tone (Nelson et al., 1995; Speers et al., 2002). All these impairments might contribute to walking disturbances, which represent a hallmark presentation of MS (Larocca, 2011). Longitudinal studies showed that up to 80% of patients with MS necessitate an assistive device for walking with disease progression (Weinshenker et al., 1989; Confavreux et al., 2000). This condition affects participation outcomes such as quality of life, daily living activities and work (Larocca, 2011).

Data from studies on healthy subjects showed that gait involves a complex interplay between cortical and spinal circuits. A full review of neural correlates of walking control is beyond this perspective. Nevertheless, the overall evidence that locomotion control relies on the integrity of feedback and feed forward mechanisms of movement control (including postural adjustments) (Dietz et al., 2002; Nielsen and Sinkjaer, 2002; Pearson, 2004) has been well established.

Although a wide range of movement control dysfunctions might contribute to gait impairment in people with MS, balance disorders are thought to contribute to most of MS walking-related disabilities. Indeed, they negatively influence gait performance by reducing gait velocity, shortening steps length, increasing double support time and decreasing single support and swing times (Cameron and Lord, 2010). It is worth noting that most of MS patients with walking disturbances report having balance problems even when they have minimal or no clinically assessable impairments (Cameron et al., 2008; Larocca, 2011; Zackowski et al., 2013).

Several studies investigating locomotion disturbances in patients with MS showed that both MS-specific reorganization of the posture control system (Corradini et al., 1997) and deficits of central integration of sensory afferents are involved as primary mechanisms. The former consists of having very delayed onset of both compensatory (CPAs) (Cameron et al., 2008; Huisinga et al., 2014) and anticipatory postural adjustment (APAs) (Krishnan et al., 2012a,b) while standing and walking. The latter consists of the inability of the central nervous system to use different sensory input (mainly vestibular, somatosensory, and visual) in order to create a system of coordinates on which the body's postural control is based (Smania et al., 2008). Central integration deficits are a rather underestimated issue in MS people even though they affect postural adjustment reactions and then balance during upright posture and gait (Huisinga et al., 2014).

As a whole, these evidence support that specific treatments aimed at improving the efficiency of postural reactions can improve gait quality and might potentially contribute to an improvement in activity, community participation, and quality of life in people with MS (Cameron et al., 2008).

Rehabilitation studies have shown that different approaches may be useful in treating balance disturbances stemming from neurological dysfunctions. Conventional balance rehabilitation strategies proved to be effective in both stroke and Parkinson disease (Smania et al., 2008, 2010). Recently, new strategies for balance rehabilitation have been put forward. On one hand, preliminary data on the effects of balance exercises stressing the processing (and/or central integration) of specific sensory afferents (i.e., somatosensory, visual, and vestibular) led to improvement of gait and balance performance in patients with stroke and spinal cord injury (Tamburella et al., 2013). On the other hand, robot-assisted training of gait in patients with Parkinson's disease has shown not only to improve gait but also balance parameters. The effects of both these approaches have been, at least in part, related to the role of somatosensory inputs in balance control. Indeed, somatic sensation (in particular proprioception) is very important for the efficiency of both feedback and feed forward control of gait and posture (Riemann and Lephart, 2002).

The aim of this study was to compare the effectiveness of end-effector robot-assisted gait training (RAGT) and sensory integration balance training (SIBT) in improving walking and balance performance in patients with MS. The hypothesis was that both types of training might promote central neural processes involved in feedback and feed forward control of gait and balance. The rationale behind the study is twofold. First, it would further explore the potential field of application of new technological devices, which are increasingly being used in clinical practice even though their mechanisms of action are still unknown. Second, it would be relevant to find new approaches which allow training patients in a safe and efficient manner even when neurological condition is severe.

## Materials and methods

### Trial design

A single blind RCT comparing the effects between the experimental (RAGT) and control group (SIBT) on walking and balance disorders was performed (allocation ratio 1:1). The examiner was blinded to group assignment.

### Participants

Outpatients with relapsing remitting or secondary progressive MS (Polman et al., 2011) were enrolled in the Neurological Rehabilitation Unit of the Department of Neurological and Movement Sciences, University Hospital, Italy. Inclusion criteria were: age 30–60 years, Expanded Disability Status Score (EDSS) between 1.5 ≥ x ≤ 6.5 (Kurtzke, 1983), Mini Mental State Examination score ≥24 (Folstein et al., 1975), ability to maintain standing position without aids for at least 1 min and ability to walk independently for at least 15 m, absence of concomitant neurological or orthopedic conditions that may interfere with ambulation. Exclusion criteria were: any type of rehabilitation intervention in the month prior to recruitment, MS relapse during the three months prior to recruitment, pharmacological therapy not well defined and/or changed during the study, presence of paroxysmal vertigo, lower limb botulinum toxin injections within the previous 12 weeks. All patients were informed regarding the experimental nature of the study. Written informed consent was given by patients. The research was performed in accordance with the Helsinki Declaration. The ethical approval was obtained from the ethics committee of the Azienda Ospedaliera Universitaria Integrata (Verona-Italy) (Prog.CE 1893). Patients enrolled in this study are a subgroup of a clinical trial registered at the http://clinicaltrials.gov (NCT01564511).

### Interventions

Prior to the start of the study, authors designed RAGT and SIBT protocols and instructed two treating physiotherapists, one for the RAGT group and the other for the SIBT group. Treatment procedures consisted of 12 individual sessions of 50 min, twice weekly (Monday and Wednesday or Tuesday and Friday) for six consecutive weeks. Both treatments were tailored to suit each patient's ability and task complexity was progressively increased as the patient improved. Patients were not allowed to receive other physiotherapy during the study, but were given no other activity restrictions. Training procedures were administered in the morning around 10 AM, to ensure that fatigue did not influence the patient's performance. Participants were allowed to wear their usual footwear and orthoses.

#### RAGT training

The RAGT group was treated by means of the electromechanical Gait Trainer GT1 (Reha-Stim, Berlin, Germany) (Hesse et al., 1999). During RAGT, individuals were secured in a harness with their feet on footplates, while movements of the center of mass were controlled in a phase-dependent manner by ropes attached to the harness. Patients received a 40 min of RAGT followed by 10 passive lower limb joint mobilizations and stretching exercises. The overall duration of RAGT therapy, including the time getting in and out was 40 min while the net RAGT lasted 30 min. Each training session consisted of two 15-min sessions, separated by a 5-min rest if required by the patient. In the first session we trained patients at 20% of supported body-weight and 1.3 km/h of speed; in the second session at 10% of supported body-weight and 1.6 km/h of speed (Picelli et al., 2012). The use of body weight support permits patients to walk more symmetrically with higher velocities resulting in a facilitation of the lower limb muscles and in a more effective gait (Hesse, 2008). In our view, the rationale for supporting body weight was to increase safety and compliance with the RAGT. Patients were instructed to “help” the GT1 gait-like movement during training. Patients unable to maintain the pace were excluded. The step-length was evaluated with the GAITRite system (CIR Systems, Havertown, PA) and individually defined (Givon et al., 2009).

#### SIBT training

The SIBT patients underwent a specific training program aimed at improving the ability to integrate multisensory inputs during balance responses (Nichols, 1997). Each session consisted of exercises fitting to three different levels of difficulty and repeated under three different sensory conditions (free vision, wearing a mask and wearing an helmet) (Smania et al., 2008). Level I included tasks that induced external destabilizations of the center-of-body mass (CoP) while standing on a stable and comfortable surface (i.e., the physiotherapist shifted the pelvis in the frontal and sagittal direction asking the patient to actively maintain balance standing on the floor). These tasks mainly involved feedback postural control. Level II included exercises of self-destabilization of the CoP. The patient performed voluntary motor actions in both static and dynamic conditions while standing on a stable and comfortable surface (i.e., performing a single-step simulation, shifting his/her weight from one foot to the other in the frontal direction standing on the floor). These tasks mainly involved feed-forward postural control. Level III consisted of exercises of external destabilization and exercises of self-destabilization of the CoP while standing on different types of compliant surfaces (i.e., increasing weight shifting and decreasing the amplitude of the base of support while standing on foam support bases of different consistency). Three different foam sections were used according to the patient's abilities (1.5, 3.5, and 8 cm). These tasks required continuous feedback and feed forward postural adjustments. During each treatment session, a total of 10 exercises (3 from level I, 3 from level II, 4 from level III) were repeated several times (2–5 times) within a 5-min period (Smania et al., 2008).

### Outcomes

An examiner, who was blinded to the patients' group allocation, performed all evaluations. Primary and secondary outcomes were measured before (T0), after treatment (T1) and at 1-month follow-up (T2). Patients were examined around 10 AM in the morning to reduce the effect of fatigue frequently reported later in the day.

#### Primary outcomes measures

***Gait speed (cm/s)***. It was assessed by the GAITRite system (Gold version 3.2b; CIR System Inc, Havertown, PA, USA) (Menz et al., 2004). Patients walk along a mat with integrated sensors 4 times in a self-selected speed. Patients were allowed to use orthoses but not other walking aids (i.e., cane).

***Berg balance scale (BBS)***. A 14-item validated scale used to evaluate both static and dynamic balance disorders after rehabilitative interventions in individuals with MS (Range of score: 0–4 points per task; higher = better performance) (Cattaneo et al., 2006).

#### Secondary outcomes measures

***Activities-specific balance confidence scale (ABC)***. A validated and reliable interview that evaluates the patient's perceived level of balance confidence during 16 daily living activities such as walking, bending, standing and reaching (Range of score: 0–100 points per activity; higher = more confident) (Powell and Myers, 1995).

***Sensory organization balance test (SOT)***. A validated balance test to evaluate central integration deficit of sensory inputs in patients with neurologic impairment. The patient stands barefoot with arms alongside the body and feet in a heel-to-toe position and maintains standing balance under 6 different sensory conditions according to the original protocol. The sensory conditions are: (1) stable surface eyes open, closed, and dome condition; and (2) compliant surface eyes open, closed and dome condition. A stopwatch records the amount of time a patient maintains erect standing without activating any postural reaction. Five 30-s trials are carried out for each condition (Range of score: 0–150 s; higher = better performance) (Shumway-Cook and Horak, 1986).

***Stabilometric assessment (SA)***. A widely used instrument to evaluate balance disorders in patients with neurological impairment. Patients are evaluated in the standing position on an electronic monoaxial platform (Technobody^©^ platform)^1^. The feet position on the platform is standardized for all patients using a V-shaped frame. The subjects while standing placed the medial borders of the feet alongside the frame. The malleolus are aligned to vertical bars. The distance between two malleolus is 3 cm and the medial borders of the feet were extra-rotated 12° with regard to the anterior-posterior axis. Patients are evaluated while standing without upper limbs support. An operator stands behind them in order to prevent the risk of falling (Cattaneo and Jonsdottir, 2009).

The patient is tested in two consecutive conditions (eyes-open and eyes-closed) each lasting 30-s according to Cattaneo and Jonsdottir's protocol (Cattaneo and Jonsdottir, 2009). Main stabilometric parameters evaluated are sway area and length of CoP trajectory.

***Fatigue severity scale (FSS)***. A 9-item self-reported questionnaire which assesses the perceived level of fatigue on a 7-point scale (Range of score: 1–7; higher = worse performance) (Krupp et al., 1989).

#### Gait analysis

The following spatiotemporal gait parameters were evaluated by means of GAITRite System: cadence (step/min), step length (cm), single support time (% of cycle) and double support time (% of cycle) (Menz et al., 2004).

***Multiple sclerosis quality of Life-54 (MSQOL-54)***. A 54-item validated structured self-report questionnaire that evaluates both generic and MS-specific domains of health-related QoL with 12 subscales. Two summary scores, physical health (PHC) and mental health (MHC) are reported (Range of score: 0–100; higher = better performance) (Solari et al., 1999).

#### Randomization procedure

After screening, an independent blinded collaborator who was not involved in the treatment or care of patients, randomly assigned eligible patients to the RAGT or SIBT according to a simple software-generated randomization scheme (Dallal, 2007).

### Statistical analysis

The Mann-Whitney test was used for testing differences between groups at baseline. The Friedman's ANOVA was used to analyse within-group changes in performance overtime, whilst the Wilcoxon signed rank tests to compare within-group changes from baseline/post-treatment and baseline/follow-up measures. The Mann-Whitney test was used for between-group comparisons. For this purpose, we computed the differences (Δ) between post- and pre-treatment performance and between follow-up and pre-treatment performance for all outcome measures. We set the alpha level for significance at 0.05, however, to adjust for multiple comparisons we used a Bonferroni correction (alpha level = 0.025). Descriptive analysis was used to evaluate the effect size measures between the 2 independent groups (Cohen's *d* calculation) (Cohen, 1988). All statistical analysis was carried out using the SPSS for Macintosh statistical package, version 16.0.

## Results

### Participants

Thirty-two patients were evaluated for eligibility between September 2011 and November 2013. Five patients were excluded because they did not meet the inclusion criteria and 1 declined to participate. Thus, 26 patients were randomly allocated to the RAGT (*n* = 14) or SIBT (*n* = 12).

**Figure 1:**
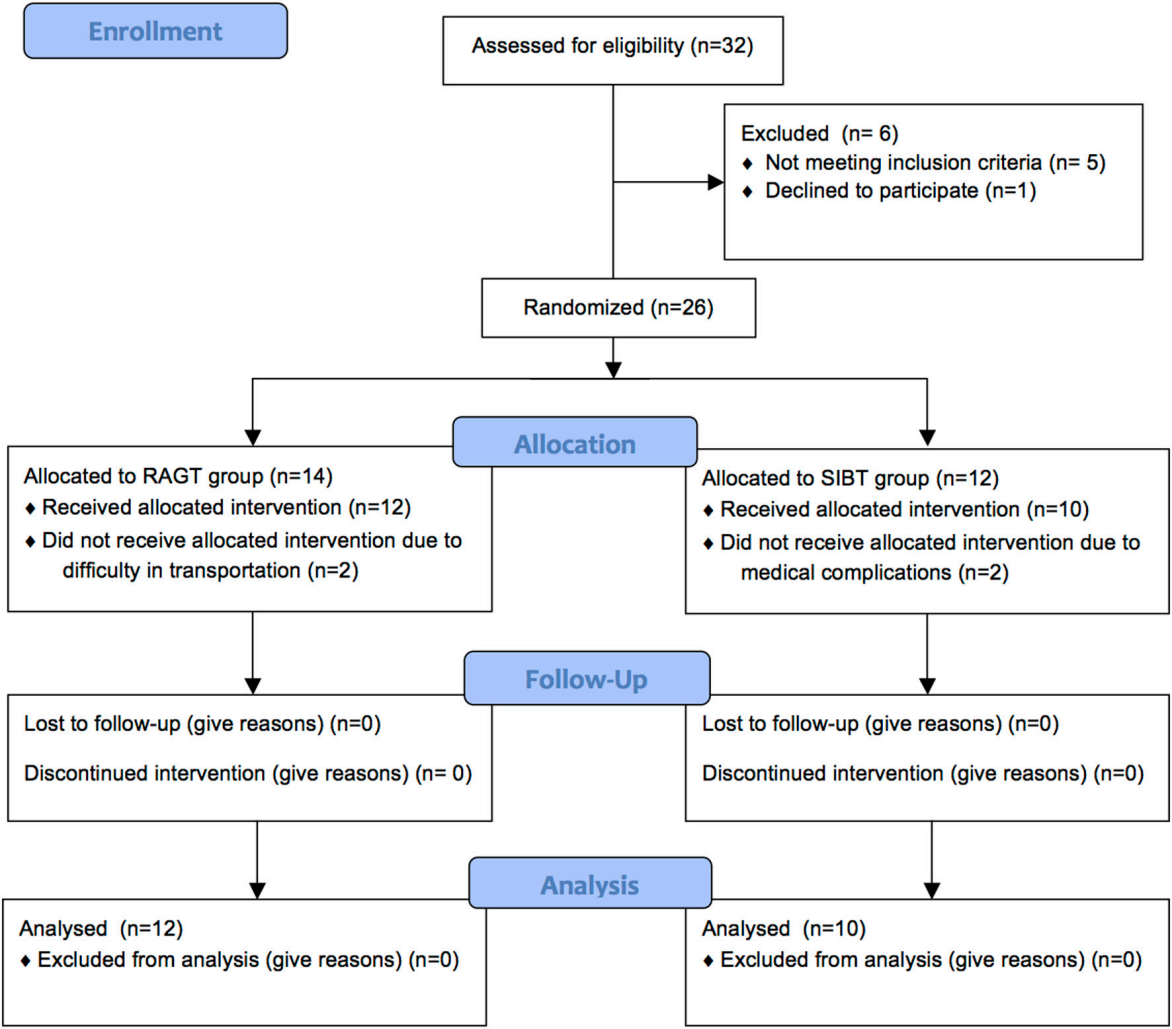
**Flow diagram of the study**.

Two patients in the RAGT and 2 in the SIBT did not complete the allocated intervention due to difficulty in transportation or medical complications to treatment sessions (Figure 1). Therefore, 12 patients in the RAGT and 10 in the SIBT completed the study (Table 1).

**Table 1:** **Baseline demographic and clinical features of the patients**.

	**RAGT group (n = 12) Mean (*SD*)**	**SIBT group (n = 10) Mean (*SD*)**	***P value* (*Z*)**
Age (years)	50.83 (8.42)	50.1 (6.29)	0.640 (−468)
Range	38–63	42–60	
Sex (Male/Female)	5/7	1/9	
EDSS	3.96 (0.75)	4,35 (0.67)	0.101 (−1.640)
Range	3–5.5	3.5–5.5	
Disease duration (years)	13.5 (7.60)	14.9 (8.68)	0.731 (−0.344)
Range	5–34	5–27	
**PRIMARY OUTCOME MEASURE**			
Gait speed (cm/s)	79.42 (21.14)	81.31 (16.81)	0.895 (−0.132)
BBS (0–56)	47.17 (5.27)	46.50 (6.69)	0.921 (−0.100)
**SECONDARY OUTCOME MEASURES**			
ABC scale (0–100)	59.68 (11.31)	61.90 (7.06)	0.226 (−1.210)
SOT S. surface (0–150)			
EO	118.73 (39.52)	114.56 (38.66)	0.691 (−0.398)
EC	63.37 (28.12)	52.23 (24.65)	0.210 (−1.253)
Dome	62.75 (40.07)	57.89 (31.97)	0.895 (−0.132)
SOT C. surface (0–150)			
EO	96.48 (37.76)	110.43 (23.27)	0.322 (−0.990)
EC	50.59 (32.95)	39.06 (16.82)	0.598 (−0.528)
Dome	58.52 (45.85)	30.96 (15.80)	0.176 (−1.352)
**Stabilometric assessment EO condition**			
Sway area (mm^2^)	83.48 (83.53)	128.54 (84.03)	0.121 (−1.550)
Length CoP (mm)	499.66 (499.0)	504.60 (408.06)	0.644 (−0.462)
**Stabilometric assessment EC condition**			
Sway area (mm^2^)	250.48 (261.99)	509.22 (342.79)	0.065 (−1.846)
Length CoP (mm)	951.66 (1045.29)	1157.90 (951.54)	0.429 (−0.791)
**Gait analysis**			
Cadence (step/min)	117.13 (28.22)	106.32 (37.49)	0.138 (−1.481)
SL (cm)	56.88 (19.57)	58.62 (25.99)	0.843 (−0.198)
SS time (% of cycle)	33.85 (4.23)	40.84 (16.49)	0.235 (−1.187)
DS time (% of cycle)	31.30 (5.48)	39.54 (14.35)	0.187 (−1.319)
FSS (1–7)	4.40 (1.386)	4.03 (2.25)	0.598 (−0.528)
MSQOL-54 PHC (0–100)	64.17 (6.53)	59.59 (10.67)	0.288 (−1.061)
MSQOL-54 MHC (0–100)	59.01 (21.69)	59.51 (20.70)	1.000 (0.000)

SD, standard deviation; P value (Z), p value and corresponding t.statistic; EDSS, expanded disability status scale; BBS, berg balance scale (higher score = better performance); ABC scale, activities balance confidence scale (higher score = better performance); SOT, sensory organization balance test; EO, eyes-open condition; EC, eyes-closed condition; S, stable; C, compliant; Length CoP, length of CoP trajectory; FSS, fatigue severity scale (higher score = worse performance); SL, step length; SS, single support time; DS, double support time; MSQOL-54 PHC, multiple sclerosis quality of Life-54 physical health (higher score = better performance); MSQOL-54 MHC, multiple sclerosis quality of Life-54 mental health (higher score = better performance); ^*^Statistically significant. p value significant if <0.05.

Multiple separate independent-sample Mann-Whitney tests showed that there was no significant difference between groups as to age, EDSS, disease duration, and all baseline clinical measures at T0 (Table 1).

#### Primary outcome measures

Between groups comparisons showed no significant changes on primary outcome measures over time (Table 2).

**Table 2:** **Descriptive and inferential statistics for clinical outcome measures**.

**Outcome variables**	**Mann-Whitney test between-group differences**	
	**T1 vs. T0 *P* value (Effect size)**	**T2 vs. T0 *P* value (Effect size)**
**PRIMARY OUTCOME MEASURE**		
Gait speed (cm/s)	0.644 (0.10)	0.895 (0.02)
BBS (0–56)	0.547 (0.13)	0.091 (0.28)
**SECONDARY OUTCOME MEASURES**		
ABC scale (0–100)	0.741 (0.09)	0.692 (0.08)
SOT—S. surface (0–150) EO	0.197 (−0.34)	0.075 (−0.37)
EC	0.947 (−0.13)	0.210 (−0.27)
Dome	0.187 (0.29)	0.553 (0.15)
SOT—C. surface (0–150) EO	0.843 (−0.05)	0.553 (0.01)
EC	0.644 (−0.12)	0.291 (−0.30)
Dome	0.129 (−0.31)	0.048 (−0.32)^*^
**SA EO condition**		
Sway area (mm^2^)	0.817 (−0.20)	0.553 (−0.10)
Length CoP (mm)	0.468 (−0.05)	0.895 (−0.10)
**SA EC condition**		
Sway area (mm^2^)	0.210 (0.20)	0.598 (0.10)
Length CoP (mm)	0.767 (0.20)	0.895 (−0.03)
**Gait analysis**		
Cadence (step/min)	0.322 (−0.22)	0.339 (−0.25)
SL (cm)	0.235 (−0.11)	0.644 (0.02)
SS time (% of cycle)	0.742 (0.15)	0.974 (−0.15)
DS time (% of cycle)	0.065 (0.41)	0.166 (0.15)
FSS (1–7)	0.276 (0.18)	0.391 (0.08)
MSQOL-54 PHC (0–100)	0.261 (−0.27)	0.869 (−0.06)
MSQOL-54 MHC (0–100)	0.667 (−0.15)	0.235 (0.07)

Before, pre-treatment; After, post-treatment; SD, standard deviation; P Value, p value; BBS, berg balance scale (higher score = better performance); ABC scale, activities balance confidence scale (higher score = better performance); SOT, sensory organization balance test; EO, eyes-open condition; EC, eyes-closed condition; S, stable; C, compliant; SA, stabilometric assessment; Length CoP, length of CoP trajectory; FSS, fatigue severity scale (higher score = worse performance); SL, step length; SS, SINGLE support time; DS, double support time; MSQOL-54 PHC, multiple sclerosis quality of Life-54 physical health (higher score = better performance); MSQOL-54 MHC, multiple sclerosis quality of Life-54 mental health;

* *Statistically significant; p value significant if <0.05*.

**Figure 2:**
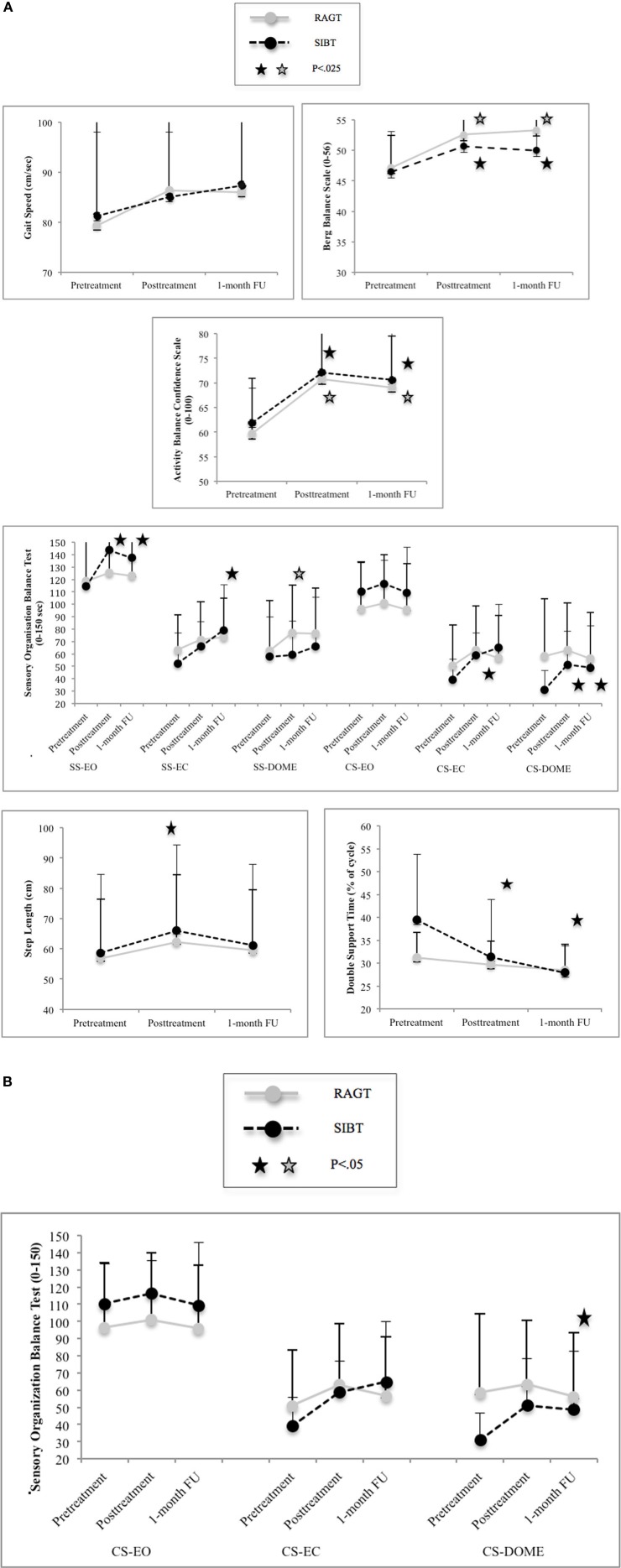
**(A)** Within group analysis: mean performance and standard errors at primary and statistical significant secondary outcome measures. Abbreviations: SS, stable surface; CS, complaint surface; EO, eyes-open condition; Dome, Dome condition; FU, follow-up. **(B)** Between group comparison: mean performance and standard errors at secondary organization balance test (SOT) (only statistical significant value). Abbreviations: CS, complaint surface; EO, eyes-open condition; EC, eyes-closed condition; Dome, Dome condition; FU, follow-up.

In the RAGT group we found within-group changes (Friedman' ANOVA) approaching significance on gait speed (*P* = 0.07) and significant changes on BBS (*P* = 0.001) over time (Figure 2). In the SIBT group overall significant changes were found only on BBS (*P* = 0.001). Pairwise comparisons are reported in Table 3A and Figure 2.

**Table 3A:** **Descriptive and inferential statistics for clinical outcome measures**.

	**Before Mean (SD)**		**After mean (*SD*)**		**1-month FU**		**Comparisons Wilcoxon signed ranks test**		**Comparisons Wilcoxon signed ranks test**	
**Outcome variables**	**RAGT**	**SIBT**	**RAGT**	**SIBT**	**RAGT**	**SIBT**	**RAGT**		**SIBT**	
							**T1 vs. T0 *P* value (95% CI)**	**T2 vs. T0 *P* value (95% CI)**	**T1 vs. T0 *P* value (95% CI)**	**T2 vs. T0 *P* value (95% CI)**
**PRIMARY OUTCOME MEASURE**										
Gait speed (cm/s)	79.42 (21.14)	81.31 (16.81)	86.49 (23.05)	85.11 (12.96)	86.04 (21.67)	87.44 (13.93)	0.117 (−1.67; 15.81)	0.050 (−2.17; 15.41)	0.515 (−8.51; 16.10)	0.333 (−6.63; 18.87)
BBS (0−56)	47.17 (5.27)	46.50 (6.69)	52.58 (2.64)	50.70 (5.74)	53.33 (2.06)	50.00 (5.46)	0.007 (1.95; 8.88)^*^	0.002 (3.07; 9.25)^*^	0.007 (1.38; 7.01)^*^	0.018 (0.38; 6.61)^*^
**SECONDARY OUTCOME MEASURES**										
ABC scale (0−100)	59.68 (11.31)	61.90 (7.06)	70.79 (11.04)	72.14 (10.24)	69.09 (10.38)	70.63 (9.72)	0.010 (2.81; 19.41)^*^	0.023 (1.88; 16.94)^*^	0.008 (3.94; 14.48)^*^	0.012 (3.67; 12.04)^*^
SOT (0−150)—S. surface EO	118.73 (39.52)	114.56 (38.66)	125.76 (34.80)	143.89 (10.88)	123.22 (35.09)	137.61 (24.38)	0.86 (−2.82; 16.88)	0.386 (−5.38; 14.37)	0.015 (0.55; 58.09)^*^	0.021 (2.49; 43.59)^*^
EC	63.37 (28.12)	52.23 (24.65)	71.53 (30.50)	65.82 (20.40)	73.83 (31.19)	79.13 (37.07)	0.136 (−7.20; 23.52)	0.209 (−10.08; 30.97)	0.047 (2.01; 25.15)	0.009 (7.70; 46.09)^*^
Dome	62.75 (40.07)	57.89 (31.97)	77.15 (38.17)	59.36 (27.36)	76.77 (36.18)	65.83 (40.28)	0.022 (1.27; 27.51)^*^	0.059 (0.30; 27.72)	0.878 (−14.54; 17.47)	0.241 (−5.58; 21.46)
SOT (0−150)—C. surface EO	96.48 (37.76)	110.43 (23.27)	100.92 (38.83)	116.56 (18.98)	95.73 (37.12)	109.46 (36.75)	0.285 (−7.10; 15.99)	0.790 (−11.97; 10.46)	0.260 (−4.13; 16.37)	0.445 (−24.38; 22.44)
EC	50.59 (32.95)	39.06 (16.82)	63.03 (35.75)	58.86 (18.41)	56.70 (34.49)	64.90 (35.38)	0.272 (−12.55; 37.43)	0.272 (−15.14; 27.36)	0.013 (6.82; 32.77)^*^	0.028 (5.10; 46.58)
Dome	58.52 (45.85)	30.96 (15.80)	63.33 (37.54)	51.33 (27.06)	56.23 (37.13)	48.87 (33.81)	0.583 (−14.19; 23.81)	0.695 (−25.61; 21.02)	0.007 (8.30; 32.43)^*^	0.009 (3.24; 32.59)^*^

Before, pre-treatment; After, post-treatment; SD, standard deviation; P Value (Z), p value; BBS, berg balance scale (higher score = better performance); ABC scale, activities balance confidence scale (higher score = better performance); SOT, sensory organization balance test; EO, eyes-open condition; EC, eyes-closed condition; S, stable; C, compliant; SA, stabilometric assessment; Length CoP, length of CoP trajectory; FSS, fatigue severity scale (higher score = worse performance); SL, step length; SS, single support time; DS, double support time; MSQOL-54 PHC, multiple sclerosis quality of Life-54 physical health (higher score = better performance); MSQOL-54 MHC, multiple sclerosis quality of Life-54 mental health;

* *Statistically significant; for post-hoc analysis p value significant if <0.025 for Bonferroni correction for primary outcome measures*.

#### Secondary outcome measures

Between groups comparisons showed significant differences on performance at SOT compliant surface-dome condition (*P* = 0.048) in favor of the SIBT training at follow-up (Table 3A and Figure 2). No significant differences between groups in all secondary outcome measures were found over time (Table 2).

In the RAGT group within-group significant changes (Friedman' ANOVA) on ABC (*P* = 0.017), on SOT stable surface eyes-closed condition (*P* = 0.04) and step length (*P* = 0.017) were found over time. Changes approaching significance were found on SOT stable surface dome condition (*P* = 0.06).

In the SIBT group within-group significant changes (Friedman' ANOVA) on ABC (*P* = 0.001), on SOT stable surface eyes-open condition (*P* = 0.013), on SOT stable surface eyes-closed condition (*P* = 0.027), on SOT compliant surface dome condition (*P* = 0.003), sway area eyes-closed (*P* = 0.04), step length (*p* = 0.002), double support (*P* < 0.001) and MSQOL-PH (*P* = 0.032) were found over time. Changes approaching significance were found on SOT compliant surface eyes-closed (*P* = 0.06) and on FSS (*P* = 0.052). Pairwise comparisons are reported in Tables 3A,B and Figure 2.

**Table 3B:** **Descriptive and inferential statistics for clinical outcome measures**.

	**Before mean (SD)**		**After mean (SD)**		**1-month FU**		**Comparisons Wilcoxon signed ranks test**		**Comparisons Wilcoxon signed ranks test**	
**Outcome variables**	**RAGT**	**SIBT**	**RAGT**	**SIBT**	**RAGT**	**SIBT**	**RAGT**		**SIBT**	
							**T1 vs. T0 P value (95% CI)**	**T2 vs. T0 P value (95% CI)**	**T1 vs. T0 P value (95% CI)**	**T2 vs. T0 P value (95% CI)**
**SECONDARY OUTCOME MEASURES**										
**SA EO condition**										
Sway area (mm^2^)	83.48 (83.53)	128.54 (84.03)	64.55 (48.67)	141.44 (124.10)	120.40 (108.76)	165.21 (133.04)	0.182 (−50.33; 12.50)	0.594 (−26.66; 60.37)	0.799 (−55.69; 81.49)	0.683 (−54.53; 127.87)
Length CoP (mm)	499.66 (499.01)	504.60 (408.06)	398.16 (354.27)	422.70 (369.64)	369.08 (342.85)	413.77 (336.63)	0.055 (−227.26; 24.26)	0.182 (−441.53; 118.84)	0.241 (−234.92; 71.12)	0.169 (−245.09; 63.44)
**SA EC condition**										
Sway area (mm^2^)	250.48 (261.99)	509.22 (342.79)	243.32 (230.54)	407.14 (311.95)	266.43 (216.58)	480.28 (330.37)	0.657 (−136.77; 122.44)	0.530 (−103.80; 135.69)	0.169 (−282.46; 78.30)	0.959 (−200.36; 142.48)
Length CoP (mm)	951.67 (1045.29)	1157.9 (951.54)	935.17 (1146.48)	1035.4 (993.74)	797.00 (736.14)	1154.78 (1053.15)	0.480 (−135.59; 102.59)	0.424 (−451.72; 142.38)	0.285 (−352.55; 107.55)	0.953 (−541.29; 304.09)
**Gait analysis**										
Cadence (step/min)	117.13 (28.22)	106.32 (37.49)	114.45 (24.75)	107.29 (42.01)	114.53 (33.78)	111.07 (40.04)	0.959 (−35.39; 11.40)	0.959 (−37.47; 13.61)	0.859 (−12.43; 14.17)	0.374 (−8.46; 17.02)
SL (cm)	56.88 (19.57)	58.62 (25.99)	62.39 (22.04)	65.95 (28.44)	59.49 (20.06)	60.99 (27.02)	0.084 (−0.32; 11.34)	0.028 (−0.00; 5.23)	0.005 (2.09; 12.56)^*^	0.508 (−3.80; 8.52)
SS time (% of cycle)	33.85 (4.23)	40.84 (16.49)	34.20 (4.39)	39.44 (16.84)	33.48 (4.93)	38.01 (10.17)	0.814 (−1.79; 2.48)	0.799 (−14.07; 2.15)	0.799 (−6.65; 3.85)	0.508 (−8.13; 2.46)
DS time (% of cycle)	31.30 (5.48)	39.54 (14.35)	29.79 (4.96)	31.40 (12.61)	28.49 (5.60)	28.03 (5.88)	0.084 (−3.42;.40)	0.074 (−16.38; 1.26)	0.005 (−15.25; −1.01)^*^	0.005 (−20.27; −2.73)^*^
FSS (1–7)	4.40 (1.38)	4.03 (2.25)	3.96 (1.17)	3.02 (1.50)	4.13 (1.81)	3.12 (1.84)	0.530 (−1.60;.73)	0.789 (−2.07;.84)	0.036 (−1.97; −0.05)	0.059 (−1.89;.07)
MSQOL−54 PHC (0–100)	64.17 (6.53)	59.59 (10.67)	60.84 (9.01)	61.34 (8.16)	60.79 (5.85)	57.30 (9.60)	0.507 (−8.04; 2.51)	0.169 (−7.20; 1.57)	0.214 (−3.60; 6.74)	0.214 (−5.75; 1.63)
MSQOL−54 MHC (0–100)	59.01 (21.69)	59.51 (20.7)	61.11 (19.58)	65.24 (15.34)	63.82 (15.01)	62.10 (18.38)	0.574 (−3.78; 7.28)	0.093 (−1.79; 9.79)	0.314 (−4.55; 14.87)	0.953 (−7.21; 11.88)

Before, pre-treatment; After, post-treatment; SD, standard deviation; P Value (Z), p value; BBS, berg balance scale (higher score = better performance); ABC scale, activities balance confidence scale (higher score = better performance); SOT, sensory organization balance test; EO, eyes-open condition; EC, eyes-closed condition; S, stable; C, compliant; SA, stabilometric assessment; Length CoP, length of CoP trajectory; FSS, fatigue severity scale (higher score = worse performance); SL, step length; SS, single support time; DS, double support time; MSQOL-54 PHC, multiple sclerosis quality of Life-54 physical health (higher score = better performance); MSQOL-54 MHC, multiple sclerosis quality of Life-54 mental health;

* *Statistically significant; post-hoc analysis p value significant if <0.025 for Bonferroni correction for primary outcome measures*.

## Discussion

Results showed that RAGT and SIBT might improve step length, postural stability and the level of balance confidence perceived while performing daily activities in patients with MS. These training effects may be maintained for at least 1 month post-treatment.

So far many approaches have been proposed to improve walking and balance in people with MS (Armutlu et al., 2001; Schuhfried et al., 2005; Newman et al., 2007; Benedetti et al., 2009; Broekmans et al., 2010; Cakt et al., 2010; Hebert et al., 2011; Swinnen et al., 2012; Nilsagård et al., 2013). However, only few RCT studies (Beer et al., 2008; Lo and Triche, 2008; Schwartz et al., 2012; Vaney et al., 2012; Straudi et al., 2013) evaluated whether RAGT may be superior to conventional walking training in terms of gait performance. Furthermore, one study of them evaluated treatment effects on mobility assessed by Time Up and Go Test (Straudi et al., 2013) and only one on balance impairment by means BBS (Schwartz et al., 2012). Beer et al. (2008) found a moderate to large effect size, although not significant, for walking speed, distance and knee-extensor strength favoring RAGT. In the present study both groups' outcomes values returned to baseline at six months follow-up (Beer et al., 2008). Lo et al. (Lo and Triche, 2008) reported in their crossover study no differences in gait outcomes between treatment groups after 6 sessions of training. Vaney et al. (2012) reported that the over ground walking group improved gait speed insignificantly more than the RAGT. Straudi (Straudi et al., 2013) and colleagues showed walking endurance, as well as spatio-temporal gait parameters improvements after RAGT. In the present study within-group analysis showed no significant effects on the TUG test (Straudi et al., 2013). Finally, Schwartz et al. (2012) revealed beneficial effects in term of gait, mobility, and balance comparable to conventional walking treatment. In the present study both RAGT and conventional walking training groups showed significant improvement on TUG test without any difference between groups (Schwartz et al., 2012). The conventional walking exercise appeared to have better long-term influence on postural control compared to RAGT in this study.

This is the first pilot study that evaluates the effects of an end-effector RAGT compared to a SIBT in walking and balance performance in patients with MS.

It is worthy to note that in all previous studies an exoskeleton device (Lokomat) was used as RAGT, and the control group consisted of over ground walking training. Thus, differences with our study were twofold. On one hand, the type of device used as RAGT was an end-effector device (Gang Trainer GT1) and on the other hand the type of treatment used as “control” condition was specific SIBT.

The Gang Trainer GT1 (Hesse et al., 1999) is an end-effector system, on which the harness-secured patients were positioned on 2 footplates, whose movements simulated stance and swing in a highly physiological manner. The body weight could be partially relieved, and ropes attached to the patient controlled the vertical and lateral movements of the center of mass in a phase-dependent manner (Hesse et al., 1999). Results showed that the GT1 training might promote changes on gait speed approaching significance. This might be attributed to the limited sample size and/or to low intensity of training procedures (30 min of RAGT, twice a week). Nevertheless, it is important to note that treatment procedures for RAGT in people with MS are still undefined in terms of intensity and variability of exercise.

Interestingly, significant changes in the GT1 group were found also on postural stability. This can be considered as one of the most important findings in our study because the majority of the existing literature on RAGT in MS patients does not evaluate this outcome. The issue of balance recovery is very relevant in MS rehabilitation studies (Cameron and Lord, 2010).

Walking can be seen as a repeated sequence of balance challenges (Cameron and Lord, 2010) and changes in gait observed in people with MS are largely the result of changes in postural control (Cameron and Lord, 2010). Overall evidence on healthy subjects showed that gait is the result of intricate dynamic interactions between a central program, so-called central pattern generator (CPG), and feedback mechanisms. The central program is based on a genetically determined spinal circuitry that allows generating basic gait functions such as starting, stopping, and steer locomotion (Rossignol et al., 2006). In contrast, feedback mechanisms activated by afferents inputs resulting from skin, muscles and special senses (vision, vestibular, and auditory) dynamically shape gait pattern to the environments necessities (Rossignol et al., 2006). To deal with these environments necessities the central nervous system employs compensatory postural adjustments or so-called feedback mechanisms (CPAs), and/or feed-forward or anticipatory postural adjustments (APAs) (Massion, 1992). Both mechanisms appear to be affected in MS patients while standing (Cameron et al., 2008) and walking (Huisinga et al., 2014).

Currently, interventions that specifically address proprioceptive and/or central processing deficits are likely to be particularly effective in MS patients. Proprioceptive information, in fact, plays a crucial role with respect to the knowledge on external environment (i.e., body position knowledge, sensorimotor control of functional joint stability and feedback postural adjustments) and in motor control during internally generated motor commands (internal model). The concept behind the study is that the task-specific balance training should improve gait performance and vice versa because postural control is essential for walking.

Our findings cannot be fully discussed with those by Straudi (Straudi et al., 2013) and Schwartz et al. (2012) owing to differences about patients EDSS score range, sample size, duration of treatment and treatment types used as both conventional and experimental treatment. In our study, balance outcome measures included also a subjective measure of confidence in performing various ambulatory activities (ABC) as well as to objective measure designed to assess static balance and fall risk (BBS). Data showed that RAGT and SIBT have a significant both post-treatment and long-term effect (1 months). Additionally, our findings suggest that these improvement may generalize in patients while performing daily activities.

A possible explanation of the balance improvements is that GT1 approach act as a form of “destabilization training.” For the first time in literature we might introduce the concept of “task specific balance training” by end-effector RAGT.

This training might play a role for reinforcing the neuronal circuits that contribute to postural control. In particular, RAGT represents an external force that could interfere with the abnormal experience of balance and gait. An end-effector system may represent a more suitable device for this purpose. It enables wheelchair-bound subjects to practice a gait-like movement with minimal assistance. The harness-secured patients are positioned on 2 footplates, whose movements simulate stance and swing in a highly physiological manner. In this context the patient has a reduced number of constraints acting at different lower limb levels. A reduced number of constraints and more freedom during exercise, especially for pelvic movements, may be an optimal environment for learning. Similar results with an end-effector system were found in patients affected by Parkinson's disease (Picelli et al., 2012, 2013). Lastly, we cannot exclude that muscle strength improvements could contribute to this effect.

This is a significant result, given that MS patients suffer from balance disorders very early during the disease even when gait disorders are minimal. From a clinical perspective having another rehabilitation strategy for these high disabling disorders is very relevant. Nevertheless future studies on larger sample and involving patients stratified by EDSS would allow us to better understand which approach (robot assisted balance training or SIBT) and for which patients would be more useful to improve balance task related domains and/or gait related domains. MS patients with different degrees of disability require different needs in terms of treatment's type, intensity, and frequency.

As to SIBT effects, significant improvements on BBS and ABC paralleled significant effects in sensory-motor integration ability and dynamic balance performance. Indeed, patients in the SIBT group showed improvements during SOT conditions (stable surface-opened eyes and closed eyes) and in most difficult performance (compliant surface-closed eyes and dome conditions). In healthy subjects balance control is a complex process involving the reception and the integration of visual, sensorimotor, and vestibular sensory inputs, which allows the planning and execution of the movements required to maintain balance during upright posture and gait (Merfeld et al., 1999). The ability of the central nervous system to process these different types of sensory information leads to the establishment of a system of coordinates on which the body's postural control is based (Merfeld et al., 1999). For instance, in the static standing position healthy adults use sensorimotor information, which originates from pressure receptors, joint receptors, and muscle proprioceptors, to build the main reference coordinates for balance (Maurer et al., 2006). When sensorimotor information is inadequate, visual and vestibular systems become involved to maintain balance. This central integration of sensory inputs allows potential sensory conflicts generated by inadequate afferent information to be overcome. The capability to analyse, compare, and select the pertinent sensory information is very important in order to prevent falling (Cattaneo and Jonsdottir, 2009). It has been showed that imbalance in MS may not only be related to a primary sensory deficit but to a disturbed integration of the available sensory information (Smedal et al., 2006). Moreover, studies of postural responses indicate that imbalance in people with MS is unlike imbalance from cerebellar disorders (Cameron and Lord, 2010).

Several studies evaluated the efficacy of rehabilitation for improving balance in people with MS (Armutlu et al., 2001; Smedal et al., 2006; Nilsagård et al., 2013). Evidence supports that the interventions most likely to be effective are those related to sensory facilitation and central integration deficits (Cattaneo et al., 2007). This type of intervention has been proposed in few studies in order to ameliorate specifically the postural control in elderly (Hu and Woollacott, 1994), in stroke patients (Smania et al., 2008; Bayouk et al., 2006) and in Parkinson disease patients (Smania et al., 2010). So far, only two RCT studies have addressed this issue suggesting promising effects in MS patients (Cattaneo et al., 2007; Elwishy, 2012). Abeer et al. enrolled fifty patients (EDSS ≤ 4.5) randomized to receive balance rehabilitation just for motor strategies or sensorimotor balance rehabilitation program that aimed at improving motor and sensory strategies. Each treatment lasted 50 min/session, 3 times/week for 8 weeks. Before and after treatment balance was assessed clinically for standing balance by BBS and instrumentally for somatosensory and neuromuscular control aspects by Biodex Stability system. Data showed significant differences in all outcome measures between the two groups in favor of the sensorimotor training (Elwishy, 2012). Cattaneo et al. (Cattaneo et al., 2007) pointed out that such sensorimotor balance training might improve also dynamic balance aspects assessed by the Dynamic Gait Index. Although differences between our work and the study by Cattaneo et al. and by Elwishy (Elwishy, 2012) were the wide variation of EDSS score, sample size, the duration of treatment and outcome measures used, our findings further support that specific balance training may induce positive effects in improving central integration of the sensory input. That is, patients underwent SIBT may have improved the ability to integrate somatosensory and vestibular inputs, becoming less reliant on visual input, as well as use the most appropriate sensory strategies to control their posture and prevent falls (Shumway-Cook and Horak, 1986; Cattaneo et al., 2007). The execution of exercises with the use of surfaces and vision manipulation aimed at challenging postural control could improve the somatosensory integration processes during dynamic tasks. This type of intervention has been proposed in few studies in order to ameliorate specifically the postural control in elderly (Hu and Woollacott, 1994), in stroke patients (Bayouk et al., 2006; Smania et al., 2008), in Parkinson disease patients (Smania et al., 2010) and in MS patients (Cattaneo et al., 2007; Elwishy, 2012).

An important finding that required discussion was the no statistically significant differences between the end-effector RAGT and the SIBT in primary outcome measures. Moreover, for improvements in secondary outcome measures, between-group differences were in favor of the SIBT for one SOT condition (compliant surface-dome). According to our hypothesis, these results support the assumption that this form of RAGT, which practices a gait-like movement with minimal assistance, allows patients to train postural and gait control. Many advantages that further support the use of end-effector RAGT may be acknowledged: the patient may be trained safely owing to body harness, the complexity of the tasks might be improved over time by changing the amount of body weight support and, finally, it does not necessarily require a one-by-one physical therapist assistance. Further, recent work has demonstrated that end-effector RAGT enables patients repetitive practice of stair climbing, which is considered a more demanding balance task than gait (Hesse et al., 2010). Stair climbing requires a high level of postural control and walking ability. It is important to consider that end-effector RAGT may allow us to develop specific programs combining gait training with sensory integration exercises in order to further improve walking. For instance by using an end-effector system (Hesse et al., 2010) with body weight support might be an optimal strategy for implementing a true sensorimotor training for people affected by MS. There is growing interest in developing new technological approaches for these disturbances.

The point to use end-effector RAGT and SIBT in people with MS is that overall evidence supports that these patients have CPAs and APAs as well as sensory integration deficits leading to “internal representation” of motor and sensory signals impairments.

People with MS have a strong delay in CPAs onset in terms of magnitude and velocity (Cameron et al., 2008; Huisinga et al., 2014). This has been attributed mainly to a reduced velocity of signals propagation in somatosensory and motor pathway. Recent findings point toward the somatosensory afferent inputs as primary causative role of impaired CPAs postural adjustment (Huisinga et al., 2014). To compensate the prolonged postural responses, and then to prevent falls, people with MS often implement a predictive strategy (APAs). Recent findings showed that APAs are impaired in people with MS in terms of delayed onset of muscles activation, reduced magnitude and less directional specific activation of muscles (Krishnan et al., 2012a,b). These alterations lead to an impaired dynamic shifting of CoP during gait initiation (Remelius et al., 2008) and to a reduction of the excursion of the center of mass in the sagittal and transversal plane (Remelius et al., 2008).

Another important mechanism involved in gait encompasses afferent input. Widespread research revealed afferent inputs are involved in motor output shaping during walking (Dietz et al., 2002; Nielsen and Sinkjaer, 2002; Pearson, 2004). It has been demonstrated that human action execution (i.e., to make a cup of coffee) required three main phases: motor planning, motor execution, and movement control. Feed-forward and feedback mechanisms are thought to be involved mainly in the last phase (movement control) in order to perform efficient goal-directed movements (Frey et al., 2011). These mechanisms are involved in forming the so-called “internal model,” which is an “internal representation” of motor and sensory signals related to a specific motor execution. It has been demonstrated that an “internal model” exists also for lower limb (Emken and Reinkensmeyer, 2005; Lam et al., 2006). It allows walking to face postural perturbation and/or novel dynamic environments. Lesions at the central nervous system due to MS can be viewed as generating a novel dynamic environment that must be learned in order to walk effectively. Experiments ranging from animals (Lou and Bloedel, 1988; Hodgson et al., 1994) to humans (Lam et al., 2003; Pang et al., 2003; Emken and Reinkensmeyer, 2005; Lam et al., 2006) showed lasting modifications in response to continuous disturbance of walking followed by aftereffects after removal the disturbance. These aftereffects that follow a period of training under specific walking condition may suggest the possible formation or adaptation of the motor output (“internal model” shaping).

Limitations of the present study are the small sample size, the clinical heterogeneity of patients according to EDSS score, the lack of patients' stratification by neurological severity, the lack of a follow-up assessment at 3 or more months after training and the lack of assessment of CPAs and APAs with electromyography. Future studies should determine frequency, duration, and other important aspects of RAGT parameters, such as speed, and need for body weight support. Finally, postural destabilizations and sensory strategies might add a substantial value to ongoing therapy.

## Tips for future research

Treatment of gait and balance dysfunction in people with MS has developed significantly in recent years. Studies demonstrated the potential effect of various interventions for improving walking and balance disorders, with benefits reported also by the patients. However, there are cloudy hypotheses that are driving this research area. To speed up the progress in this field of research, several crucial points should taken into account when planning future studies (Zackowski et al., 2013):

1. To perform randomized controlled trial on larger sample in order to evaluate SIBT and end-effector RAGT effectiveness in MS patients. In our opinion, the RAGT devised for this purpose should be an end-effector one in order to improve gait and balance too.
2. To evaluate the effects of treatments combining SIBT and end-effector RAGT.
3. To develop new technological software that may include for instance exercises with sensory augmentations for the impaired proprioception and/or with the use of surfaces and vision manipulation aimed at improving somatosensory integration processes (Elwishy, 2012), sub sensory mechanical noise or vibration applied to the sole of the foot, haptic learning (without vision) and functional electrical stimulation cycling to coordinate further leg muscles during walking.
4. To couple visual information and feedback in order to improve the awareness of disturbed walking and to engage actively the patients' participation and motivation during training.
5. To amplify the patient's movement errors (Emken and Reinkensmeyer, 2005) aimed at inducing postural adaptations and favorably adjust leg movement trajectories. Unexpected perturbation during swing phase of walking might be useful (Emken and Reinkensmeyer, 2005).

## Conclusions

This is the first study comparing the effects on walking and balances between the end-effector RAGT and SIBT. These preliminary results suggest that the end-effector RAGT training may act as task-specific balance training in order to promote central neural processes involved in gait and balance control.

### Conflict of interest statement

The authors declare that the research was conducted in the absence of any commercial or financial relationships that could be construed as a potential conflict of interest.

## Footnotes

^1^Website: http://www.tecnobody.it.
